# Fermentation Profile, Aerobic Stability, and Chemical and Mineral Composition of Cactus Pear Silages with Different Inclusion Levels of Gliricidia Hay

**DOI:** 10.3390/plants13020195

**Published:** 2024-01-11

**Authors:** Moema Kelly Nogueira de Sá, Alberício Pereira de Andrade, Gherman Garcia Leal de Araújo, André Luiz Rodrigues Magalhães, Cleyton de Almeida Araújo, Roberta de Lima Valença, Amélia de Macedo, Antônia Rafaela da Silva Oliveira, Anderson de Moura Zanine, Daniele de Jesus Ferreira, Fagton de Mattos Negrão, Thieres George Freire da Silva, Fleming Sena Campos, Glayciane Costa Gois

**Affiliations:** 1 Programa de Pós-Graduação em Ciência Animal e Pastagens, Universidade Federal do Agreste de Pernambuco, Garanhuns 55292-270, Brazil; moemaa.sa@gmail.com (M.K.N.d.S.); albericio3@gmail.com (A.P.d.A.); andre.magalhaes@ufape.edu.br (A.L.R.M.); rafacosta2@live.com (A.R.d.S.O.); 2 Setor de Produção Animal, Empresa Brasileira de Pesquisa Agropecuária, Embrapa Semiárido, Petrolina 56302-970, Brazil; ghermangarcia@hotmail.com; 3 Programa de Pós Graduação em Ciência Animal, Universidade Federal do Vale do São Francisco, Petrolina 56310-770, Brazil; alcleytonaraujo@gmail.com (C.d.A.A.); ameliamacedo71@gmail.com (A.d.M.); 4 Departamento de Zootecnia, Universidade Federal do Espírito Santo, Alegre 29500-000, Brazil; robertalimav@hotmail.com; 5 Programa de Pós-Graduação em Ciência Animal, Universidade Federal do Maranhão, Chapadinha 65500-000, Brazil; anderson.zanine@ufma.br (A.d.M.Z.); daniele.ferreira@ufma.br (D.d.J.F.); flemingcte@yahoo.com.br (F.S.C.); 6 Departmento de Zootecnia, Instituto Federal de Educação, Ciência e Tecnologia de Rondônia, Colorado do Oeste 76993-000, Brazil; fagton.negrao@ifro.edu.br; 7 Programa de Pós-Graduação em Produção Vegetal, Universidade Federal Rural de Pernambuco, Serra Talhada 56909-535, Brazil; thieres.silva@ufrpe.br

**Keywords:** ensiling, forage preservation, *Gliricidia sepium*, *Opuntia ficus* indica Mill, semi-arid

## Abstract

Cactus pear is used in large proportions in diets for small ruminants in semiarid regions. However, its exclusive use is not recommended due to the low fiber and crude protein content and the high water and mineral content, leading to metabolic disorders, low dry matter intake, and weight loss. The use of mixed cactus silage associated with protein and fibrous sources seeks to overcome the deficits in dry matter, fiber and crude protein, aiming to improve the nutritional quality of the diets that will be offered to ruminants. Thus, the use of gliricidia hay in cactus pear silages could represent an important alternative to improve the nutritional and fermentative characteristics of the ensiled material. Therefore, our aim was to evaluate the fermentation dynamics, nutritional characteristics, and aerobic stability of mixed silages of cactus pear combined with different levels of gliricidia hay. This was a completely randomized experimental design with five treatments and five repetitions. The treatments consisted of different levels of inclusion of gliricidia hay (0, 10, 20, 30, and 40% on a dry matter basis) in the composition of mixed cactus pear silages. The inclusion of gliricidia hay in the composition of mixed silages of cactus pear resulted in a quadratic effect for dry matter recovery, pH, NH_3_-N, buffering capacity, aerobic stability, ether extract, P, K, Na, and Zn (*p* < 0.05). There was a reduction in density, effluent losses, maximum pH, mineral matter, non-fiber carbohydrates, Ca, Mg, Fe, and Mn (*p* < 0.05), and an increase in the time to reach maximum pH as well as an upward trend in pH, dry matter, organic matter, crude protein, neutral detergent fiber, acid detergent fiber, and B (*p* < 0.05). Under experimental conditions, the inclusion of gliricidia hay between 20 and 30% in cactus pear-based silage provided an improvement to the chemical composition and fermentation parameters of the silages.

## 1. Introduction

One of the main obstacles to animal production in dryland regions is underfeeding due to limitations related to the availability of food and water, leading to low net income for livestock farmers. This is more pronounced during the dry season of the year [1], making dietary supplementation necessary in this period. However, herd supplementation increases production costs. In this sense, aiming to ensure sustainable animal production, feeding strategies for herds in periods of forage scarcity have been analyzed and adopted to meet the animals’ production requirements throughout the year, in an economical and viable way in the semi-arid context [2].

Using appropriate technologies such as forage preservation as silage is an alternative to overcoming problems due to food shortages in dryland regions [3]. Cactus pear silage (*Opuntia ficus* indica Mill.) is a food alternative for use in arid and semi-arid regions, as, in addition to providing nutritional support, it can also reduce the water needs of animals [4].

Cactus pear is a plant that presents morphophysiological characteristics that allow it to adapt to the soil and climate conditions of the semi-arid region, including high production potential per unit area, high nutritional value and water content, and even meeting some of the water needs of herds [5]. However, due to its high water content and low contents of dry matter, fiber, and protein, when making silages, cactus pear must be combined with other ingredients to complement its nutritional composition and promote good functioning and maintenance of the rumen microbiota [6] when composing diets for ruminants.

Therefore, the combination of cactus pear with other species of forage plants adapted to the semi-arid region in the form of hay, in the preparation of mixed silages, could represent an important alternative to improve the nutritional and fermentation characteristics of the ensiled material [4]. In this context, gliricidia hay (*Gliricidia sepium*) can be used as an absorbent additive and increase the dry matter, crude protein, and physically effective fiber contents of cactus pear silages, improving the fermentation and nutritional characteristics of cactus pear silages and increasing the efficiency of the ensiling process [7].

Hypothesizing that gliricidia hay ensures the preservation and improves the nutritional quality and fermentation profile, reducing fermentation losses of cactus pear silage, the aim of this study was to evaluate the fermentation dynamics, nutritional characteristics, and aerobic stability of mixed silages of cactus pear combined with different levels of gliricidia hay.

## 2. Results

### 2.1. Fermentation Losses and Fermentation Profile

The inclusion of gliricidia hay in the composition of mixed cactus silages involves density (*p* = 0.001) and effluent losses (*p* < 0.001) in silage with the inclusion of gliricidia hay. A quadratic effect was obtained for dry matter recovery (*p* = 0.014), pH (*p* < 0.001), NH_3_-N (*p* = 0.010), and TC (*p* = 0.012) as the levels of gliricidia hay in mixed cactus silages increased (Table 1).

### 2.2. Aerobic Stability

Gliricidia hay reduced the maximum silage pH (*p* = 0.018) and increased the time to reach maximum pH (*p* = 0.010), and the trend of pH also increased (*p* = 0.001) in cactus pear silages. There was a quadratic effect on the time to reach the maximum silage temperature (*p* < 0.001), the maximum difference between silage temperature and ambient temperature (*p* = 0.009), the sum of the difference between the silage temperature and ambient temperature (*p* = 0.001), and aerobic stability (*p* = 0.002) with increasing levels of gliricidia hay in mixed silages of cactus pear (Table 2).

### 2.3. Chemical Composition

The inclusion of gliricidia hay in cactus pear silages increased the contents of dry matter (*p* < 0.001), organic matter (*p* < 0.001), crude protein (*p* < 0.001), neutral detergent fiber (*p* < 0.001), neutral detergent fiber corrected for ash and protein (*p* < 0.001), and acid detergent fiber (*p* < 0.001), but reduced the contents of mineral matter (*p* < 0.001) and non-fiber carbohydrates (*p* < 0.001). There was a quadratic effect on ether extract (*p* < 0.001) as the levels of gliricidia hay included in cactus pear silages increased. There was no effect of gliricidia hay inclusion levels on the total carbohydrate content (*p* > 0.05) (Table 3).

### 2.4. Mineral Composition

The inclusion of gliricidia hay in the composition of mixed silages of cactus pear caused a quadratic effect on the contents of phosphorus (*p* = 0.012), potassium (*p* = 0.007), sodium (*p* = 0.001), and zinc (*p* = 0.001). There was a reduction in calcium (*p* < 0.001), magnesium (*p* < 0.001), iron (*p* < 0.001), and manganese (*p* < 0.001), but an increase in the boron content (*p* < 0.001) with increasing levels of gliricidia hay in the composition of mixed silages. There was no effect of the inclusion of gliricidia hay on the sulfur or copper contents (*p* > 0.05) (Table 4).

## 3. Discussion

### 3.1. Fermentation Losses, Fermentation Profile, and Aerobic Stability

The inclusion levels of gliricidia hay tested in this study were not sufficient to increase the densities in cactus pear silages to levels between 600 and 800 kg/m^3^, which are considered adequate for well-compacted silages [8]. This is related to the higher dry matter content of gliricidia hay (90.57% natural matter; Table 1), which made the silage compaction process difficult, causing a reduction in density. According to Borges et al. [9], silages with densities below 600 kg/m^3^ result in a greater volume of residual air in the mass, which leads to a higher release of CO_2_ and loss of DM.

However, despite not obtaining density values within the literature’s specifications, the inclusion of gliricidia hay in silages reduced effluent losses compared to the control silage (silage of cactus pear alone). Thus, gliricidia hay is inferred to favor the fermentation process, reducing losses and favoring the dry matter recovery of silages. Corroborating our findings, Brito et al. [10] reported a reduction in fermentation losses with increased dry matter recovery when including different levels of gliricidia in cactus pear silages.

The combination of cactus pear and gliricidia hay in the composition of mixed silages contributed to the increase in pH from 4.35 (10% gliricidia hay) to 4.62 (40% gliricidia hay). This result was related to the buffering capacity of gliricidia, which, like all legumes, has high levels of orthophosphate and organic acid salts besides the high protein content [11]. The pH values found in silages were similar to those reported by Brito et al. [10] in silages of cactus pear with gliricidia (3.68–4.96). According to Pahlow et al. [12], silages with dry matter content between 30 and 50% may have pH values between 4.35 and 5.00 and remain stable after fermentation, which may have occurred in this study. In cactus pear and elephant grass silages, Santos et al. [13] observed that increasing the density of the silage improved its fermentation profile as a result of the drop in pH. However, the authors inferred that silages with a higher proportion of cactus pear than the forage plant used as a moisture-absorbing additive do not allow for compaction similar to that achieved with other forage plants due to the risk of higher effluent losses, with a consequent pH above 4.2.

The NH_3_-N content found in silages is an indirect indicator of clostridial activity and can contribute to increases in silage pH [14]. In this study, the NH_3_-N content varied between 4.31 and 5.73%, with higher values in silages that had higher pH values due to the higher inclusion of gliricidia hay in their composition. Such results are considered desirable because they are below 10% [15], which indicates no excessive breakdown of proteins to ammonia, characterizing proper silage fermentation. According to Zanine et al. [16], NH_3_-N below 10% is acceptable as it does not cause intoxication and it improves the voluntary intake of silage by animals.

As legumes have a high protein content, low soluble carbohydrate content, and high buffering capacity, the buffering capacity of the silages evaluated was expected to increase with the increasing inclusion of gliricidia hay in cactus pear silages. However, this did not occur, so the lowest BC was found in silage with the highest proportion of gliricidia hay (40%). The reduction in BC could be related to the composition of cactus pear, which has high content of minerals such as calcium (22.15 g/kg DM), potassium (45.65 g/kg DM), and magnesium (6.89 g/kg DM) [17], which have buffering activity, neutralizing the organic acids formed by fermentation and preventing a drop in pH [15]. Furthermore, the reduction in silage moisture caused by the inclusion of hay in the ensiled mass may also have contributed to the reduction in the buffering capacity of the silage, which is beneficial because, according to Muck [18], the higher the buffering capacity, the greater the amount of lactic acid that will have to be formed so that the pH reaches levels sufficient to inhibit the activity of clostridial microorganisms and enterobacteria, which harm the silage quality.

The exposure of silages to the aerobic environment reduced the maximum pH registered, and the inclusion of hay increased the time required for the mass to reach the maximum pH. This effect is beneficial for maintaining the nutritional quality of the silage during exposure to air, as aerobic microorganisms use residual fermentation substrates and organic acids from the fermentation process for colonization and population increases in an aerobic environment [19]. With the inclusion of gliricidia hay up to 20% in cactus pear silages, the TMT increased by approximately 8.4 h compared to the silage of cactus pear alone, with a higher DTS also being observed, resulting in a greater heating capacity of the silage (2.4 °C) and shorter time to break aerobic stability (72 h). This production of heat from the silage indicates that the aerobic deterioration process is beginning, and losses of dry matter will occur due to the oxidation of the material [20].

As the increase in temperature is related to the dry matter content of the silage [21], this effect demonstrates that the increase in silage DM promoted an increase in osmotic pressure, which reduced the activity of harmful microorganisms, improving its quality [15]. However, for the inclusion of gliricidia hay at levels above 20% in cactus pear silage, a reduction in temperature and DTS was observed, as also reported by Araújo et al. [22] when *Arachis pintoi* was included in elephant grass silage. These authors found that the inclusion of up to 20% *Arachis pintoi* in silages caused an increase in the temperature and DTS of the silages and associated this result with the reduction in oxygen during fermentation, which tends to reduce the temperature of silages.

When the silo is opened, the exposure to oxygen causes an increase in temperature due to exothermic reactions such as respiration and multiplication of microorganisms, which degrade the silage [23]. Although the lowest AE was at the level of 20% inclusion of gliricidia hay, all silages showed good fermentation patterns, with aerobic stability above 70 h. This was only achieved by Brito et al. [10] with the inclusion of 75% gliricidia in cactus pear silage, confirming the use of gliricidia hay as a good additive and improving the fermentation quality of cactus pear silage.

### 3.2. Chemical Composition

The dry matter content of the material to be ensiled is essential for the ensiling process, as it determines the type of fermentation that will develop inside the silo [24]. Therefore, although gliricidia hay acts as a moisture sequester, cactus pear contains mucilage, which is composed of glycoprotein and organic acids, providing it with water retention capacity [25]. In this sense, although gliricidia hay increases the dry matter content of silages, only silages containing 30 and 40% gliricidia hay have a matter content between 30 and 35%, which is recommended by McDonald et al. [15] to achieve good fermentation in the silo.

The reduction in mineral matter occurred because cactus pear naturally has higher concentrations of this component than gliricidia hay. This allowed for the regulation of the osmotic potential, making it more negative than the environment, thus enabling water absorption [26]. Consequently, when adding gliricidia to the mixture, the contribution of minerals and moisture from the cactus pear decreased, which explains why the mineral matter contents decreased and the dry matter increased.

The increase in the crude protein content of the ensiled mass is the main advantage of mixed silage production. In this way, it is possible to observe the beneficial effect of the combination of gliricidia hay with cactus pear in the composition of mixed silages, which increased the crude protein content from 5.427% (silage of cactus pear alone) to 18.549% (silage containing 40% gliricidia hay). The crude protein contents in the silages were above the minimum necessary to ensure proper rumen fermentation without compromising the efficient use of fiber carbohydrates. According to NRC [27], when the crude protein content in feed offered to small ruminants is below 7%, there is low availability of N, which can reduce fiber digestion and intake due to the slow passage of food through the rumen. Adequate contents of crude protein serve as an indication of lower intensities of proteolysis during the fermentation of the ensiled material [28] and improve the development of rumen flora and the fermentation process, enabling an increase in the rate of passage of nitrogenous material to the small intestine [14].

Similar to the crude protein content, the ether extract of the silages increased with the addition of gliricidia hay to their composition. As cactus pear has a higher ether extract content than gliricidia hay, it is possible that the higher proportion of cactus pear in the silage composition than the hay levels tested contributed to these results. According to Marques et al. [29], ether extract below 5% is beneficial so that feed intake by ruminants is not limited. Therefore, the combination of these roughages in the composition of mixed silages was inferred to have balanced the energy value of the silage [22]. The silages tested here can be used to feed ruminants as they allow for a higher intake by the animals and because they do not present limitations due to excess fiber, low protein concentration, or high energy concentration [30].

Higher neutral detergent fiber and acid detergent fiber contents in gliricidia hay contributed to the increase in these components in silages. Despite the observed increase, the neutral detergent fiber and acid detergent fiber values obtained in all silages studied were below the maximum limit for neutral detergent fiber (60%) and acid detergent fiber (40%) recommended by Van Soest [31] regarding diets for small ruminants. These improve the digestibility and intake of food by animals by facilitating the colonization of the feed by rumen microorganisms, which, in turn, can induce higher fermentation rates [32].

The increase in fiber fractions can lead to a reduction in total carbohydrates and non-fibrous carbohydrates, a fact observed here with increasing levels of gliricidia hay in silages and by Borges et al. [9] when including buffel grass hay in cactus pear silages. In this sense, it is clear that the nutritional composition of silage depends on the concentrations of nutrients from the forage plants used to make the silages and that the synchronization between the nutrients present in gliricidia hay and cactus pear, mainly the amount of fiber carbohydrates, non-fibrous carbohydrates, and proteins, can provide the animal with adequate rumen kinetic conditions, resulting in greater efficiency in the development of rumen microorganisms [33].

### 3.3. Mineral Composition

Along with the fermentation process, which generates losses of minerals through percolation, the levels of K, Ca, and Mg were reduced with the inclusion of gliricidia hay in silages because these minerals are present in a higher proportion in cactus pear than in hay. The results found here are above those required for the daily intake of beef cattle, which is 3–4 g/kg K, 1.54 g/kg Ca, and 1 g/kg Mg, according to body weight, following the recommendations from the NRC [34].

Due to the higher P content in gliricidia (3.2–4.9 g/kg) [35] compared to cactus pear (1.24–1.54 g/kg [17]), this nutrient was expected to be increased with increasing levels of inclusion of gliricidia hay in the silage composition, compared to the control silage (0% hay). However, despite the increase in P levels, the silages would not meet the needs of beef cattle (1.6 g/kg) [34], which would require dietary supplementation.

According to Cunha et al. [36], cactus pear contains a high proportion of Na due to its Crassulacean acid metabolism. In the present study, the silage of cactus pear alone presented a Na value higher than that presented by silages containing gliricidia hay in its composition, meeting the requirements of beef cattle (0.6–0.8 g/kg) [34] for this mineral. The high Na content highlights the importance of silage of cactus pear alone for ruminant nutrition, as this mineral plays a key role in maintaining osmotic pressure, acid–base balance, and control of water metabolism [19]. In addition, Na can reduce feed intake because it increases animal thirst; however, as cactus pear is also a source of water for ruminants, this unfavorable effect of Na is probably minimized.

The increase in B content in the tested silages is associated with the element content in gliricidia hay (53 mg/kg) [37]. No research has evaluated the Boron content in legume–cactus silages combined with gliricidia, just as there are no recommendations for the Boron content in the composition of diets to meet the nutritional requirements of beef cattle [38]. According to Araújo et al. [17], the increase in B content is important because this micronutrient brings benefits to the immune system, with a direct action on the thyroid and Ca metabolism, and has effects on reproductive activity in males.

The high Fe contents found in silages are above the maximum recommended for beef cattle (500 mg/kg) [38]. As the inclusion of gliricidia hay reduced the Fe content in cactus pear silages, studies to evaluate the levels of gliricidia hay above 40% in the composition of mixed silages with cactus pear are necessary so that adequate Fe levels can be obtained to meet the demands of cattle. Wysocka et al. [39], in a study on Fe in cattle health, found that excess Fe can affect animal performance, interfering with the use of Cu, P, Zn, and Mg. Furthermore, Fe has a mutual absorption pathway with Mn, competing for transferrin binding sites. Therefore, when there is a reduction in Fe, there is an increase in the absorption of Mn in the animal organism [40]. In this study, a reduction in the Mn content of silages was observed with the inclusion of gliricidia hay; however, the Mn values were within the tolerable limit for cattle diets (maximum tolerable concentration of 1000 mg/kg) [38].

The importance of Zn in animal organisms is due to the involvement of this microelement in the synthesis of vitamin A, in the transport of CO_2_, in the degradation of collagen, in the metabolism of carbohydrates, in the destruction of free radicals, and in the stability of the erythrocyte membrane [41]. Gliricidia hay provided an increase in this nutrient in silages compared to the silage of cactus pear alone; however, all silages presented Zn values that met the requirements of beef cattle (30 mg/kg) [38].

Due to the importance of minerals in animal nutrition and the few studies that have evaluated these nutrients in the ingredients that will make up the diets offered to ruminants, more studies are needed to clarify the existing gaps.

## 4. Materials and Methods

### 4.1. Experimental Site

The experiment was conducted at Embrapa Semiárido, in Petrolina, state of Pernambuco, Brazil (latitude 9°8′8.9″ S, longitude 40°18′33.6″ W, altitude 373 m). The region is characterized by a BSwh semi-arid climate, characterized by scarce and irregular rainfall and strong evapotranspiration as a result of high temperatures. During the experimental period, the average temperature, relative humidity, and evapotranspiration were 25.51 °C, 63.87%, and 3.51 mm, respectively.

### 4.2. Experimental Design

This was a completely randomized experimental design with five treatments and five repetitions. The treatments corresponded to the inclusion of different levels of gliricidia hay (0, 10, 20, 30, and 40% on a dry matter basis) in the composition of mixed silages with cactus pear. 

### 4.3. Silage-Making

For the silages, cactus pear of the Mexican elephant ear variety was harvested manually 24 months after regrowth. Gliricidia was harvested six months after planting, with an average height of 1.5 m. Plants were cut 30 cm above the ground, and the upper thirds of the plants with young leaves and more tender stems were harvested. Gliricidia haymaking was carried out in the field, where the harvested material was placed on a tarp and left to dry for 48 h, being turned over twice during the process. All material was chopped in a stationary forage machine (PP-35, Pinheiro Máquinas, Itapira, São Paulo, Brazil) to 2.0–2.5 cm particles. Samples of the chopped material were collected for analysis (Table 5).

The material was homogenized manually according to the treatments. After homogenization, ensiling was carried out in experimental silos (10 × 50 cm) equipped with Bunsen valves to allow for gas escape. Two kilograms of sand were deposited at the bottom of the experimental silos, protected by a thin plastic screen and a layer of non-woven fabric (TNT), preventing the ensiled material from coming into contact with the sand and allowing the effluent to drain. After closing, the silos were stored for 45 days in a covered warehouse.

### 4.4. Silage Fermentation Losses

After 45 days, the silos were weighed and opened. Density (D; kg/m^3^), effluent losses (EL; kg effluent/ton green matter), and dry matter recovery (DMR; %) were estimated according to Zanine et al. [42] using the equations below:

D = m/V(1)

where m = weight of the ensiled material expressed in kg and V = volume of the ensiled material.

After removing all forage from the experimental silo, the empty set was weighed and, subtracting from this the weight of the set before ensiling, the effluent production was estimated:

EL= {WSEo − WSEc/FMc} × 1000(2)

where WSEo = weight of the silo when empty upon opening (kg); WSEc = weight of the silo when empty upon closing (kg); FMc = forage mass upon closing (kg).

The dry matter recovery (DMR) was carried out according to the difference in weight, which was obtained by weighing the forage mass at the times of ensiling and opening and their respective DM contents:

DMR = ((FMo × DMFo)/(FMc × DMFc)) × 100(3)

where FMo = forage mass in the opening (kg); DMFo = dry matter content of forage upon opening (%); FMc = forage mass upon closing (kg); DMFc = dry matter content of forage upon closing (%).

### 4.5. pH and Ammonia Nitrogen (NH_3_-N)

Samples were taken upon silo opening to determine the pH and ammonia nitrogen (NH_3_-N). The pH levels of the samples were measured using a portable digital pH meter (Marconi^®^ MA-552, Piracicaba, SP, Brazil), which had been previously calibrated. For the analysis of ammonia nitrogen in the silages, 25 g silage samples were weighed and placed in 250 mL containers containing 200 mL of sulfuric acid solution (H_2_SO_4_; 0.2 N). Then, each container was sealed and allowed to rest under refrigeration for a period of 48 h. After this resting period, the material was filtered through filter paper. Immediately after this procedure, 1.5 mL of the filtrate was removed and subjected to centrifugation at 13,000 rpm for 10 min. Subsequently, the supernatant was transferred to eppendorfs and 10 μL of this material was pipetted into a test tube. Then, 1.5 mL of phenol solution was added. This solution was stirred using a vortex.

Subsequently, 1.5 mL of sodium hypochlorite solution and 1.5 mL of sodium hydroxide were added to this solution and stirred again with the help of a vortex. After this procedure, the tubes were taken to a water bath, remaining for 15 min at 39 °C. Subsequently, readings were taken using a spectrophotometer. The results were expressed in relation to total nitrogen (NH_3_-N/TN) [43].

### 4.6. Aerobic Stability

To determine aerobic stability (AE, h), the methodology adapted by Araújo et al. [44] was used. All procedures were carried out in a closed room, under controlled temperature conditions. The temperature of the silage mass was measured upon experimental silo opening using a digital infrared thermometer (Benetech, Rio de Janeiro, RJ, Brazil). The internal temperatures (T, in °C) of the silages were measured at intervals of 1 h for 120 h. The pH levels of the silages were determined during aerobic stability every 6 h, for 96 h of exposure to air. The following were determined: time to reach maximum pH (maximum TpH, h); trend of pH increase (TE pH; h); maximum temperature (MT; °C); time to reach maximum temperature (TMT; h); maximum difference between silage temperature and ambient temperature (DST; °C); sum of the maximum difference between silage temperature and ambient temperature (∑DT; °C); and aerobic stability.

### 4.7. Buffering Capacity

The buffering capacity (BC; e.mg NaOH/100 g DM) was determined by following the methodology of Playne and McDonald [45]. A silage sample approximately 20 g in size was diluted with 250 mL distilled water and titrated with HCl (0.1 N) until reaching pH 3.0 to release bicarbonates as carbon dioxide. Subsequently, titration was carried out with NaOH (0.1 N) until pH 6.0. The buffering capacity was determined by the equation below:

BC = (0.1 × (Va − Vb) × 100)/DSW(4)

where 0.1 = NaOH normality; Va = volume of NaOH used to titrate the sample to obtain pH 6.0; Vb = volume of NaOH used to titrate the blank to obtain pH 6.0; DSW = dry sample weight = [(sample weight × DM)/100].

### 4.8. Chemical and Mineral Composition

Samples of the material before and after silo opening were pre-dried in a forced ventilation oven at 55 °C for 72 h and processed in a knife mill (Wiley mill, Marconi, MA-580, Piracicaba, Brazil) using 1 mm sieves. Samples were analyzed for their contents of dry matter (DM, method 930.15), mineral matter (MM, method 942.05), organic matter (OM), crude protein (CP, method 984.13), ether extract (EE; method 920.29), acid detergent fiber (ADF, method 973.18) [46], neutral detergent fiber (NDF) [47], neutral detergent fiber corrected for ash and protein (NDFap) [48], total carbohydrates (TC) [49] and non-fiber carbohydrates (NFC) [50].

The concentrations of the minerals phosphorus (P), potassium (K), calcium (Ca), magnesium (Mg), sodium (Na), sulfur (S), boron (B), copper (Cu), iron (Fe), manganese (Mn), and zinc (Zn) were determined according to Araújo et al. [17].

### 4.9. Statistical Analysis

The data were subjected to the Shapiro–Wilk and Levene tests to verify the normality of residuals and homogeneity of variances, respectively; once the assumptions were met, the data were analyzed using the PROC GLM procedure from SAS University Software (SAS University) and tested by analysis of variance and regression at the level of 5% probability of type I error. The significance of the parameters estimated by the models and the coefficients of determination were used as selection criteria for the regression model. The following statistical model was used:

yij = µ + Ti + εij(5)

where μ = overall mean; Ti = effect of including gliricidia hay; εij = residual error.

## 5. Conclusions

The inclusion levels of 20 and 30% gliricidia hay in cactus pear silage resulted in a better fermentation profile of the silage, with an increase in the contents of dry matter, crude protein, and nutrients. Our findings evidence the potential of gliricidia hay to be used as a new alternative in animal feed in the Brazilian Northeast region, even providing better efficiency through the use of cactus pear plantations, which could result in a reduction in labor. However, for a better nutritional characterization, future studies that evaluate the content of organic acids, carbohydrates, and protein fractionation of the silages evaluated are necessary and pertinent.

## Figures and Tables

**Table 1:** Fermentative losses and fermentative profiles of mixed cactus silages with different levels of gliricidia hay.

Items	Gliricidia Hay Levels (%)					SEM	*p*-Value	
	0	10	20	30	40		L	Q
Density (kg/m^3^) ^1^	528.90	461.67	441.05	437.48	392.00	9.99	0.001	0.062
Effluent losses (kg/t natural matter) ^2^	67.84	31.26	4.55	3.14	5.14	4.26	<0.001	<0.001
Dry matter recovery (%) ^3^	89.79	94.36	94.30	89.11	85.93	2.05	0.060	0.014
pH ^4^	4.65	4.35	4.42	4.49	4.62	0.03	0.489	<0.001
Ammonia nitrogen (NH_3_-N/total nitrogen) ^5^	5.73	4.32	4.31	4.88	5.28	0.39	0.778	0.010
Buffering capacity (E.mgNaOH/100 g dry matter) ^6^	82.09	89.84	71.15	57.33	50.44	2.37	<0.001	0.012

SEM = standard error of the mean; *p*-value = probability value; L = linear effect; Q = quadratic effect. Equations: ^1^ ŷ = 541.62 − 29.799x, R^2^ = 0.89; ^2^ ŷ= 156.303 − 12.177x + 0.216x^2^, R^2^ = 0.94; ^3^ ŷ = 84.274 + 7.544x − 1.474x^2^, R^2^ = 0.90; ^4^ ŷ 4.618 − 0.024x + 0.0006x^2^, R^2^ = 0.80; ^5^ ŷ = 5.579 − 0.123x + 0.003x^2^, R^2^ = 0.83; ^6^ ŷ = 85.847 − 0.260x − 0.017x^2^, R^2^ = 0.88.

**Table 2:** Aerobic stability of mixed cactus silages with different levels of gliricidia hay after 45 days of ensiling.

Items	Gliricidia Hay Levels (%)					SEM	*p*-Value	
	0	10	20	30	40		L	Q
Maximum pH ^1^	5.48	5.25	5.01	4.6	5.01	0.19	0.018	0.151
Maximum TpH (h) ^2^	36.0	78.0	78.0	72.0	96.0	12.75	0.010	0.389
TE pH (h) ^3^	18.0	34.8	22.8	32.4	33.6	2.40	0.001	0.298
Maximum temperature (°C)	23.60	24.40	24.20	23.80	24.00	0.20	0.755	0.076
TMT (h) ^4^	2.80	4.80	10.4	9.20	6.40	1.04	0.049	<0.001
DTS (°C) ^5^	1.20	1.60	1.80	1.00	1.00	0.16	0.073	0.009
∑DT (°C) ^6^	−26.2	−5.2	2.4	−17.4	−9.8	3.81	0.103	0.001
Aerobic stability (h) ^7^	>96.0	85.6	72.0	>96.0	>96.0	4.69	0.177	0.002

Maximum TpH = time to reach maximum pH; TE pH = trend of pH increase; TMT = time to reach maximum temperature; DST = maximum difference between silage temperature and ambient temperature; ∑DT = sum of the maximum difference between silage temperature and ambient temperature; SEM = standard error of the mean; *p*-value = probability value; L = linear effect; Q = quadratic effect. Equations: ^1^ ŷ = 5.392 − 0.0158x. R^2^ = 0.59; ^2^ ŷ = 49.200 + 1.140x, R^2^ = 0.66; ^3^ ŷ = 22.560 + 0.288x, R^2^ = 0.37; ^4^ ŷ = 2.057 + 0.584x − 0.012x^2^, R^2^ = 0.84; ^5^ ŷ = 1.263 + 0.0414x − 0.00129x^2^, R^2^ = 0.62; ^6^ ŷ = −23.103 + 1.754x − 0.039x^2^, R^2^ = 0.51; ^7^ ŷ = 95.863 − 1.620x + 0.046x^2^, R^2^ = 0.57.

**Table 3:** Chemical composition of mixed cactus silages with different levels of gliricidia hay after 45 days of ensiling.

Items (g/kg Dry Matter)	Gliricidia Hay Levels (%)					SEM	*p*-Value	
	0	10	20	30	40		L	Q
Dry matter (g/kg natural matter) ^1^	97.57	176.81	258.14	329.41	408.87	3.57	<0.001	0.480
Mineral matter ^2^	273.14	184.14	162.27	153.78	133.28	6.26	<0.001	<0.001
Organic matter ^3^	750.75	828.79	849.19	856.45	874.84	5.59	<0.001	<0.001
Crude protein ^4^	54.27	119.73	130.87	167.27	185.49	4.64	<0.001	0.001
Ether extract ^5^	12.53	13.46	16.62	16.66	15.57	0.89	<0.001	<0.001
Neutral detergent fiber ^6^	242.89	298.77	328.68	333.21	367.19	9.33	<0.001	0.061
NDFap ^7^	224.38	277.15	307.44	316.48	359.26	1.13	<0.001	<0.001
Acid detergent fiber ^8^	183.20	244.93	257.11	272.83	275.04	13.04	<0.001	0.047
Total carbohydrates	667.23	682.66	690.19	662.27	665.64	7.69	0.345	0.052
Non-fibrous carbohydrates ^9^	442.85	405.50	382.75	345.79	306.37	7.41	<0.001	0.516

SEM = standard error of the mean; *p*-value = probability value; L = linear effect; Q = quadratic effect. Equations: ^1^ ŷ = 97.748 + 8.027x, R^2^ = 0.99; ^2^ ŷ = 264.824 − 7.397x, R^2^ = 0.94; ^3^ ŷ = 757.920 + 6.543x, R^2^ = 0.94; ^4^ ŷ = 69.534 + 3.100x, R^2^ = 0.93; ^5^ ŷ = 5.726 + 0.853x − 0.015x^2^, R^2^ = 0.98; ^6^ ŷ = 257.544 + 2.83x, R^2^ = 0.92; ^7^ ŷ = 235.126 + 3.091x, R^2^ = 0.95; ^8^ ŷ = 206.745 + 1.994x, R^2^ = 0.70. ^9^ ŷ = 443.186 − 3.326x, R^2^ = 0.99.

**Table 4:** Mineral composition of mixed cactus silages with different levels of gliricidia hay after 45 days of ensiling.

Items	Gliricidia Hay Levels (%)					SEM	*p*-Value	
	0	10	20	30	40		L	Q
	macrominerals (g/kg dry matter)							
Phosphorus ^1^	0.99	1.30	1.46	1.26	1.24	0.102	0.160	0.012
Potassium ^2^	20.84	15.40	12.80	12.35	13.75	1.48	0.001	0.007
Calcium ^3^	55.61	33.56	25.91	23.50	21.07	1.14	<0.001	<0.001
Magnesium ^4^	5.18	5.14	4.74	3.97	3.74	0.25	<0.001	0.833
Sulfur	2.18	2.38	2.40	2.56	2.57	0.24	0.997	0.215
	microminerals (mg/kg dry matter)							
Sodium ^5^	927.70	495.70	260.20	263.70	324.20	89.48	<0.001	0.001
Boron ^6^	33.30	65.88	66.95	74.88	77.17	3.28	<0.001	<0.001
Copper	18.27	20.92	21.30	18.16	20.49	1.48	0.723	0.458
Iron ^7^	1762.07	1091.14	832.19	602.78	577.35	135.94	<0.001	0.013
Manganese ^8^	75.43	50.20	39.92	39.15	31.88	3.15	<0.001	<0.001
Zinc ^9^	44.97	54.96	53.30	53.50	50.52	1.78	0.095	0.001

SEM = standard error of the mean; *p*-value = probability value; L = linear effect; Q = quadratic effect. Equations: ^1^ ŷ = 1.017 + 0.033x − 0.0007x^2^; R^2^ = 0.82; ^2^ ŷ = 20.735 − 0.624x + 0.011x^2^; R^2^ = 0.99; ^3^ ŷ = 54.112 − 2.062x; R^2^ = 0.97; ^4^ ŷ = 5.316 − 0.0405x; R^2^ = 0.94; ^5^ ŷ = 916.957 − 49.361x + 0.874x^2^; R = 0.99; ^6^ ŷ = 36.617 + 2.502x; R = 0.91; ^7^ ŷ = 1544.670 − 2.858x; R = 0.85; ^8^ ŷ = 66.796200 − 0.973750x; R = 0.81; ^9^ ŷ = 46.086 + 0.784x − 0.017x^2^; R = 0.80.

**Table 5:** Chemical composition of cactus pear and gliricídia hay before ensiling.

Items g/kg Dry Matter	Cactus Pear	Gliricidia Hay
Dry matter (g/kg natural matter)	82.13	905.73
Mineral matter	251.19	103.96
Crude protein	52.13	187.96
Ether extract	30.80	11.95
Neutral detergent fiber	206.74	327.15
Acid detergent fiber	117.97	217.33
Hemicellulose	88.77	109.82
Lignin	40.62	87.14
Non-fibrous carbohydrates	432.53	348.15
Total carbohydrates	665.89	696.22

## Data Availability

Data are contained within the article.
